# Advancements in Imaging for Atrial Fibrillation Ablation: Is There a Potential to Improve Procedural Outcomes?

**DOI:** 10.19102/icrm.2020.110701

**Published:** 2020-07-15

**Authors:** Edmond Obeng-Gyimah, Saman Nazarian

**Affiliations:** ^1^Perelman Clinical Electrophysiology Section, Cardiovascular Division, Department of Medicine, School of Medicine at the University of Pennsylvania, Philadelphia, PA, USA

**Keywords:** As low as reasonably achievable (ALARA), computed tomography, electroanatomic mapping, image integration, intracardiac echocardiography, late gadolinium enhancement, magnetic resonance imaging

## Abstract

Since the introduction of atrial fibrillation (AF) ablation in the 1990s, the procedure has continuously evolved, with gradual improvements in outcomes and safety. Recent technological advancements include the introduction of contact force catheters and high-resolution electroanatomical mapping systems, while imaging modalities including transesophageal echocardiography and fluoroscopy have become integral parts of AF ablation procedures. Further, intraprocedural intracardiac echocardiography and the integration of cardiac magnetic resonance and computed tomography images with electroanatomical mapping have shown promise to improve procedural outcomes by reducing radiation exposure and procedural times. However, available data on procedural utility and the reduction in AF recurrence rates associated with these modalities are mixed. This review therefore aims to discuss the current common imaging modalities used in AF ablation and their potential impact on outcomes. In particular, imaging is discussed with respect to the important information it offers before, during, and after the procedure. Perspectives on the future of imaging in AF ablation are also shared.

## Introduction

Atrial fibrillation (AF) is the most common form of arrhythmia in the United States and around the world.^1^ Current estimates suggest the number of patients afflicted with AF exceeds 30 million worldwide and three million in the United States.^2^ The economic burden of AF has been clearly established.^3^ The mainstay of AF management includes the prevention of stroke and the introduction of rhythm control strategies involving either antiarrhythmic drugs or catheter ablation. Since the introduction of AF ablation in the 1990s,^4^ the procedure has continuously evolved, with gradual improvements in both outcomes and safety. Recent technological advancements include the introduction of contact force catheters and high-resolution electroanatomical mapping systems, while imaging modalities including transesophageal echocardiography and fluoroscopy have become integral parts of the AF ablation procedure. Similarly, the introduction of intraprocedural intracardiac echocardiography and the integration of cardiac magnetic resonance and computed tomography images with electroanatomical mapping have shown promise in improving procedural outcomes by reducing radiation exposure and procedural times. However, available data on procedural utility and the reduction in AF recurrence rates associated with these modalities are mixed. This review aims to discuss the current common imaging modalities used in AF ablation and their potential impact on outcomes. In particular, imaging is discussed with respect to the important information it offers before, during, and after the procedure. Perspectives on the future of imaging in AF ablation are also shared.

AF ablation is effective at improving quality of life and symptoms, particularly in cases where antiarrhythmic drugs have failed.^5^ Techniques have evolved over recent decades with substantial improvements in safety and procedure outcomes. Current guidelines recommend ablation for paroxysmal AF refractory to class I or III antiarrhythmic drugs.^5^ Furthermore, given the improving safety profile and efficacy, there is a class IIa recommendation to conduct ablation prior to the initiation of antiarrhythmic drugs and for persistent AF.^5^ Several imaging modalities have been adopted to assist the electrophysiologist with the ablation procedure, each of which assists with different aspects of the procedure, with the overall aim of improving the outcome, safety, or radiation exposure. For instance, transesophageal echocardiography (TEE) is typically employed to rule out left atrial (LA) appendage (LAA) thrombus pre-ablation, intracardiac echocardiography (ICE) enhances the safety of transseptal puncture and catheter tissue contact during ablation, cardiac magnetic resonance imaging (MRI) and computed tomography (CT) help to integrate chamber and pulmonary venous anatomy into the procedural electroanatomic map, and CT imaging alone may be used to rule out atrioesophageal (AE) fistula following ablation if concerning symptoms are noted. Regardless of the imaging modality being used, however, it is important that the electrophysiologist balances the additional information with costs and real value in terms of procedure safety, radiation exposure, and outcomes.

### Preprocedural imaging

One of the most important aspects of AF management is the prevention of LAA thrombus formation by the institution of oral anticoagulation. The incidence of stroke among patients undergoing pulmonary vein (PV) isolation (PVI) has been estimated to be approximately 1.5%.^6^ Data from the surgical literature suggest that 57% of thrombi in rheumatic AF and 91% of thrombi in nonrheumatic AF are identifiable in the LAA.^7^ Current guidelines for imaging to rule out LAA thrombus before ablation remain similar to those for electrical cardioversion, with the presence of LAA thrombus being a contraindication to the procedure. The gold-standard modality for the identification of LAA thrombus is TEE.^5,8^ In comparison with TEE, CT imaging showcases excellent negative predictive value but poor specificity and positive predictive value, although the specificity could significantly improve to 99% with delayed CT.^8^ Cardiac MRI has also been tested for the identification of LAA thrombus in comparison with TEE, with some studies^9,10^ concluding that it, especially when using delayed enhancement sequences, shows comparable performance to that of TEE in identifying LAA thrombi. Advantages of cardiac MRI over CT include a lack of iodinated contrast and radiation exposure. However, CT offers significantly higher spatial resolution, albeit with lower temporal and contrast resolution relative to cardiac MRI. Notably, CT and particularly cardiac MRI are prone to motion artifacts that are often encountered in the cardiac population with arrhythmia and difficulty with breath-holds. In comparison with CT and cardiac MRI, TEE is invasive and associated with an approximately 0.9% risk of complications such as esophageal perforation, bleeding, sedation-related events, arrhythmia, and even death.^10,11^ For patients unable to undergo TEE due to swallowing difficulties or other gastrointestinal problems, cardiac MRI and CT represent viable options for ruling out LAA thrombus prior to ablation. In contrast, TEE is portable and does not use iodinated or gadolinium contrast agents. However, it is important to note that multiple recent studies have reported that the risk of nephrogenic systemic sclerosis with gadolinium chelates is exceedingly low, especially when new macrocyclic and linear agents are used.^12^ Recently, phased-array ICE has also emerged as a potential adjunct to TEE for LAA thrombus detection. A 2014 study^13^ found that ICE was equally as good as TEE and exhibited potential advantages in identifying LAA thrombus. The best imaging outcome for LAA thrombus with ICE is usually obtained with the ICE catheter located in the pulmonary artery. However, manipulation in the pulmonary artery potentially increases the risk of perforation and requires proficiency with ICE manipulation; thus, more studies are required for further validation of the safety and efficacy of this technique.^5^

**Figure 1:**
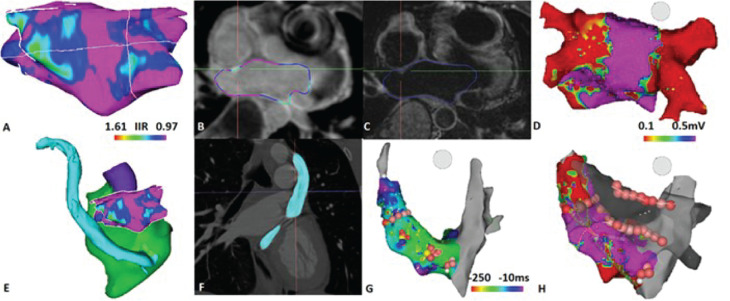
Examples of the utility of image integration for AF ablation. **A:** CMR-segmented left atrial shell [posteroanterior (PA) view]. The image intensity ratio (IIR) was used to define atrial scar (IIR > 1.61 correlates with < 0.1 mV; IIR < 0.97 correlates with ; 0.5 mV). The patient underwent prior PVI five months before imaging. **B:** High-resolution LGE cardiac MRI image (axial view). An atrial scar is noted at the bilateral pulmonary vein antra. **C:** T2-space cardiac MRI image (axial view). Better identification of the atrial wall of the left atrium is apparent. **D:** Before ablation, a prior ablation scar at the bilateral PV antra is observable (voltage map, PA view). **E:** Display of CMR-segmented left atrial scar shell and CT-segmented left-sided SVC. **F:** Left-sided SVC on CT angiography. **G:** Non-PV triggers at the ostium of the coronary sinus, proximal coronary sinus, and left-sided SVC (ablation lesions on non-PV triggers, activation map). **H:** Final ablation lesions on the posterior wall, non-PV triggers, and left-sided SVC isolation (voltage map of left-sided SVC).

Aside from identifying LAA thrombi, preprocedural imaging can provide important anatomic and structural information to assist with the procedure. Reviewing PV anatomy, presence of a patent foramen ovale, anomalous PV drainage or left persistent superior vena cava (SVC), LA size, and LA scar and fibrosis **(Figure 1)** is important for optimal procedure planning. A lack of detailed understanding of variabilities in PV structure and branching could result in incomplete isolation of all veins or PV stenosis. The most common variations of PV anatomy described in the literature include left common PV ostium (observed upwards of 83% of the time), right common PV ostium (seen in up to 40% of cases), and separate ostium for the right middle vein (affecting up to 27% of cases).^14^ In a study by Toffanin et al. evaluating PV anatomy with TEE and magnetic resonance angiography (MRA), only 42% of patients showed normal PV anatomy with two right and left veins.^15^ Measuring the PV diameter size could be essential in procedure planning for cryoablation and is best performed with three-dimensional (3D) imaging modalities such as CT or cardiac MRI. TEE appears to be better able at identifying a patent foramen ovale when compared with CT or cardiac MRI. TEE can also evaluate PV anatomy well, achieving up to 95% concordance with MRA.^15^ Although TEE tends to underestimate ostial measurements,^16^ image integration with electroanatomic mapping requires expertise and can be limited by inaccurate segmentation and image registration. Cardiac MRI is comparable to CT in the evaluation of the PVs^17^ before AF ablation. Moreover, cardiac MRI remains the gold standard for evaluating fibrosis and scar. Late gadolinium enhancement (LGE) cardiac MRI images of the LA, used to evaluate fibrosis before ablation, have been shown to also predict AF recurrence following ablation.^18,19^ However, it is important to note that the methods to analyze the extent of LGE vary significantly across centers and result in variable readouts. We have identified and validated two image-intensity standardization methods, image-intensity ratio and z-score which may enhance the homogeneity of data analysis across multiple centers.^20,21^

Other predictors of AF recurrence include LA diameter and volume measurements collected before ablation. These could be obtained readily prior to the ablation procedure with transthoracic echocardiogram (TTE), TEE, CT, or cardiac MRI. Parikh et al. evaluated the performance of LA diameter and volumes using TTE, TEE, and CT.^22^ The study only used diameter measurements from TTE but adopted volume measurements from both TEE and CT. The results confirmed that a larger LA, as measured by either diameter or volume, predicted the recurrence of AF postablation. The use of TEE diameter measurements was more effective than those from TTE and adoption of the TEE volume was superior to the diameter measurement in predicting AF recurrence. Further, however, CT LA volume assessment was superior to TEE volume assessment in predicting AF recurrence.^22^

### Intraprocedural imaging

#### Image integration

At the time of AF ablation, CT, cardiac MRI, or intraprocedural ICE images can be integrated and registered onto the electroanatomic and/or fast activation map. This practice has been shown to reduce procedural and fluoroscopic times and may improve outcomes. In a study that randomly assigned patients undergoing AF ablation (from 2005–2007) to either image integration with CT and electroanatomic mapping using the CartoMerge system (Biosense Webster, Diamond Bar, CA, USA) versus the traditional method without integration,^23^ the AF-free survival rate in the integration group was much higher over 14 months ± 12 months of follow-up (88% versus 69%; p = 0.017). A subsequent publication by Bertaglia et al. confirmed the superiority of image integration.^24^ In another registry study using data from patients with paroxysmal AF undergoing ablation in 12 Italian centers, Bertaglia et al. reported improved outcomes with regard to procedure duration and recurrent atrial arrhythmias postablation when image integration with electroanatomic mapping was performed preablation.^24^ Interestingly, fluoroscopic times are generally not lower in such studies; in fact, some studies have reported higher levels of X-ray exposure in the image integration group,^23^ whereas others showed no difference.^24^ It is important to emphasize that these findings have not been consistent. When Caponi et al. examined outcomes postablation in a randomized controlled manner, no difference in disease recurrence or complications was noted when image integration was used.^25^ However, there was a reduction in fluoroscopic duration correlated with image integration. The introduction of fast anatomical mapping and multipolar catheters, which occurred after the majority of the above studies were performed, has further led to decreased utilization of image registration during AF ablation. It is notable, however, that, in the identification of smaller early PV branches, data on proximity to external structures of interest and regions with late enhancement are not provided with fast anatomical mapping. Additionally, the smoothing algorithms of fast anatomic mapping software often create errors at the PV ostia, which have to be subsequently “erased.” Without great attention to detail, such errors can direct lesions away from intended ostial targets.

When compared with preprocedural CT or cardiac MRI, intraprocedural ICE offers real-time information such as catheter feedback or the development of effusion prior to clinical manifestations in heart rate and blood pressure and can even identify thrombus formation on catheters during ablation.^26,27^ Furthermore, registration errors from volume shifts are avoided with ICE. ICE has also been shown to improve procedural outcomes and reduce complications in AF ablation.^28^ However, ICE is more likely to miss small proximal branches from PVs, which may be prone to stenosis, as well as other PV anomalies. Thus, we believe a combination of segmented cardiac MRI and live ICE alongside electroanatomic mapping provides the most comprehensive and valuable set of data during AF ablation.

The effects of radiation exposure in interventional cardiology are well-established.^29,30^ The guiding principle for radiation use in the interventional laboratory, endorsed by major organizations including the Centers for Disease Control and Prevention and the American College of Cardiology, goes by the acronym ALARA (“as low as reasonably achievable”). Further studies are still needed to clearly establish that image integration with preprocedural cardiac MRI reduces the total amount of radiation exposure. Additionally, the use of ICE and electroanatomic mapping can significantly reduce or even eliminate fluoroscopy, as detailed below. It is clear, however, that the total radiation applied to the patient will be increased when preprocedural CT is performed.

#### Zero-fluoroscopy ablation

Given the mandate of ALARA, the concept of zero fluoroscopy in AF ablation is gaining traction. It is generally accepted that zero radiation is better than any radiation and that no radiation dose is considered safe for the patient, the electrophysiologist, or the cardiac catheterization laboratory staff in general. Orthopedic injuries among interventionalists and electrophysiologists are also well-documented.^31^ Thus, the avoidance of fluoroscopy is appealing provided that the operator is comfortable with adopting alternative guidance methodologies for all procedural components. Electroanatomic mapping and ICE are the main imaging modalities employed for this purpose. The feasibility of zero fluoroscopy was initially shown in 2010. In the study, 20 patients with paroxysmal AF underwent ablation and were followed up with after zero-fluoroscopy AF ablation using the EnSite™ NavX™ mapping system (Abbott Laboratories, Chicago, IL, USA). Transseptal puncture was guided by ICE, while catheter advancement from the lower vessels into the heart was managed by EnSite™ NavX™ electroanatomic map guidance. Some patients already had CT imaging segmented and integrated with the EnSite™ NavX™ maps, thus improving procedure time. The overall procedure time was longer than that which would be expected for routine AF ablation but, after over six months of follow-up, only 10% of cases experienced recurrence and all had isolated veins.^32^ Most importantly, there were no complications reported in this first zero-fluoroscopy AF ablation study. Meanwhile, other groups have recently shared their workflow for zero-fluoroscopy AF ablation using the CARTO^®^ mapping system (Biosense Webster, Diamond Bar, CA, USA)^29^ and image integration with preprocedural CT or cardiac MRI or intraprocedural 3D ultrasound reconstruction of the LA. Besides the feasibility of zero-fluoroscopy AF ablation procedures, some recent studies have shown that the efficacy, overall procedure duration, and radiofrequency ablation time are not impaired.^33^

#### Electroanatomic mapping with the use of fast anatomic mapping or image integration during atrial fibrillation ablation

The benefits of electroanatomic mapping during AF ablation with respect to X-ray exposure and procedural time have been established. A prospective randomized trial in 2005 performed by Rotter et al. showed clearly that procedure duration and X-ray exposure were reduced by the technology^34^ and their findings have been confirmed by subsequent studies.^35^ An important aspect of most mapping system platforms is the ability to superimpose voltage or activation information upon segmented, registered, and integrated cardiac MRI or CT images, which can enhance procedural guidance. Visualization of abnormal LA bipolar voltage, usually defined as less than 0.5 mV, has been associated with extensive LGE on cardiac MRI^20^ and separately with the failure of AF ablation and increased recurrence rates.^36,37^ Other studies have reported improved outcomes when low-voltage areas, as identified by voltage maps, are targeted for ablation.^38,39^ Large, prospective randomized trials are lacking to confirm improved outcomes in AF ablation based on substrate ablation using voltage mapping with and without the aid of image integration.

#### Other imaging modalities at the time of atrial fibrillation ablation

Image integration with preprocedural CT or cardiac MRI segmentation of the esophagus may be limited in directing ablations away from the esophagus due to small location changes from esophageal peristalsis.^40,41^ Additionally, volume shifts may contribute to errors with electroanatomic map registration. Rotational angiography in the electrophysiology (EP) suite addresses inaccuracies in registration by obtaining X-ray images with the C-arm immediately prior to ablation. These images are 3D-segmented and superimposed either on fluoroscopic images or on the electroanatomic map. Some studies have reported reduced radiation doses inherent with rotational angiography in comparison with multislice CT imaging.^42^ However, to circumvent the above problems and to enhance real-time feedback during the procedure while eliminating ionizing radiation, the concept of real-time cardiac MRI for EP procedural guidance was introduced in 2008.^43^ The major advantages of real-time cardiac MRI include providing real-time information on lesion formation and supporting a reduction in fluoroscopy; however, the technology of real-time cardiac MRI in the EP laboratory is still in its infancy. Several centers have reported conducting simple atrial flutter ablations under cardiac MRI guidance^44^ but significant improvements in workflow and noise and image artifact control with suitable electrogram recordings, mapping, and ablation in the strong electromagnetic field associated with this imaging modality have to be resolved before its wide acceptance can proceed.

### Postprocedural imaging

The main purpose of postprocedural imaging in AF is to monitor complications and/or help predict recurrence. The incidence of critical pericardial effusion postablation ranges from 1% to 1.3%.^45^ In the immediate postprocedural period, ICE could be instrumental in assessing for cardiac perforation and pericardial effusion from ablation. Not so infrequently, patients may be hypotensive immediately after ablation due to sedation changes or as an immediate reaction to protamine for the reversal of anticoagulation. If access is still maintained, ICE can quickly reassure the operator that any finding of hypotension is not due to pericardial effusion. If sheaths have already been removed, then TTE is a viable choice that could rule out effusion with outstanding accuracy. Another possible complication post-AF ablation is phrenic nerve injury, which has an approximate frequency of 1% with radiofrequency ablation and that of 5% with cryoablation. In the immediate postoperative period, fluoroscopy with or without a “sniff test” could show elevated hemidiaphragm if concerns about phrenic nerve injury exist.

One of the most dreaded complications of AF ablation is AE fistula. Although rare, with a reported incidence rate of 0.03% to 0.08%, it is catastrophic, leading to a greater than 55% mortality rate in patients who experience the complication.^46^ When AE fistula is not recognized or is addressed only with conservative management, mortality is high. Han et al. showed in a meta-analysis that the median time to presenting with symptoms is approximately 21 days but could range from the day of the procedure to as long as 60 days after the procedure. The symptoms could be variable, with a majority of affected patients presenting with infectious symptoms. Abdominal pain and cardiac symptoms are also common. The imaging modality of choice for evaluating AE fistula is CT with contrast. Echocardiography, including TTE and TEE, should be avoided when AE fistula is suspected given the high false-negative rates and clinical deterioration, including strokes, reported when TEE was performed.^46,47^ With prompt identification and surgery, the mortality rate could be improved to approximately 33%, as shown in the largest reported series of cases of AE fistula.

PV stenosis is another serious complication that can occur after AF ablation. Prior to the advent of wide antral circumferential ablation, PVI consisted mostly of ostial ablation, with a risk of PV stenosis upwards of 42%.^48^ With current technology and wide antral ablation, this risk has been reduced significantly to approximately 0.3% to 3.4%.^48,49^ If PV stenosis is suspected after the procedure, cardiac MRI, CT, PV angiography, and TEE are all viable options for diagnosis.^50^

Another potential utility for imaging following AF ablation is to help predict recurrence and the regions of reconnection after PVI. Cardiac MRI studies have shown the regional lack of LGE to predict regions of reconnection during the second procedure after initial cryoablation with reasonable accuracy.^51^ Mishima et al. showed that, when LGE cardiac MRI was performed in patients with recurrent AF after initial ablation, regions without scar predicted accurately, in 93% of cases, where reconnections would be noted during EP study at the time of the second procedure. Other research suggests the sensitivity of cardiac MRI seems to be quite limited for gap identification after radiofrequency ablation^52^ and its utility to be minimal following cryoablation.^53^ The extent of LGE on cardiac MRI has been shown to predict ablation success.^54^ McGann et al. performed cardiac MRI before, immediately after, and at three months after AF ablation procedures. Dark, nonenhanced regions noted immediately following the procedure, suggesting no reflow, correlated with locations of scarring three months after surgery and predicted procedural success. Meanwhile, regions of hyperenhancement seen immediately after the procedure represented a continuum of inflammation to necrosis and did not correlate as well with scar at three months of follow-up relative to nonenhanced regions. The nonenhanced regions were shown to demonstrate the no-reflow phenomenon, representing coagulation as well as contraction necrosis.

## Summary

In this review, a variety of image modalities used in AF ablation procedures have been presented. Fluoroscopy, image integration with electroanatomical mapping, and ICE are important adjuncts for AF ablation. Image integration provides the electrophysiologist with detailed anatomic roadmaps with the potential to reduce fluoroscopic exposure and procedural times and possibly improve outcomes. The integration of cardiac MRI, in particular, is advantageous given the lack of radiation exposure or iodinated contrast use in addition to information regarding the presence and amount of fibrosis, which may be crucial for the selection of patients and targets for ablation. With recent studies reporting efficacy and procedure duration outcomes comparable to those of traditional methods, zero fluoroscopy will likely gain a significant foothold in the context of AF ablation. The advantages of no fluoroscopy imparted to the patient, staff, and the operator are obvious as no fluoroscopy has been deemed safe and orthopedic injuries to operators in lead aprons are well-documented. Larger randomized controlled trials are needed to definitively answer the question of whether advanced imaging improves procedural success rates in AF. The eagerly anticipated DECAAF II study, which explores whether ablating fibrotic regions improves freedom from AF, is expected to significantly add to our understanding of the importance of advanced imaging in AF ablation.
